# Effects of sodium‐glucose cotransporter type 2 inhibitors on cardiovascular, renal, and safety outcomes in patients with cardiovascular disease: a meta‐analysis of randomized controlled trials

**DOI:** 10.1186/s12933-021-01272-z

**Published:** 2021-04-22

**Authors:** Caiyun Zheng, Meimei Lin, Yan Chen, Haiting Xu, Lingqun Yan, Hengfen Dai

**Affiliations:** 1 Affiliated Fuqing City Hospital of Fujian Medical University, No. 267 Qingrong Avenue, Fuqing, Fuzhou, 350300 Fujian China; 2 Fujian Medical University, No. 1 Xueyuan Road, University Town, Fuzhou, 350122 Fujian China; 3 Affiliated Fuzhou First Hospital of Fujian Medical University, No. 190, Dadao Road, Taijiang District, Fuzhou, 350009 Fujian China

**Keywords:** Sodium glucose cotransporter type 2 inhibitors, Cardiovascular disease, Mortality, Meta‐analysis

## Abstract

**Background:**

Controlled studies and observational studies have shown that sodium-glucose cotransporter type 2 inhibitors (SGLT-2i) are beneficial for the survival of patients with heart failure (HF). However, it is unclear whether SGLT-2i can provide benefit in patients with other cardiovascular diseases. Here, we conducted a systematic review and meta-analysis to determine the outcomes of cardiovascular, renal, and safety outcomes of SGLT-2i administration in patients with cardiovascular diseases.

**Methods:**

We searched PubMed, EMBASE, Cochrane Library, Web of Science databases, and ClinicalTrials.gov databases for randomised controlled trials written in English from inception until November 1, 2020. Two reviewers independently identified randomised controlled trials comparing the effects of SGLT-2i in patients with cardiovascular disease with or without diabetes. Primary outcomes were cardiovascular outcomes and renal outcomes. Secondary outcomes were safety outcomes, including adverse endocrine outcomes and adverse infection outcomes. The effects of SGLT-2i were evaluated using RevMan5.3 software. The Cochrane risk of bias tool was used to assess study quality.

**Results:**

We identified 10 randomised controlled trials (25,108 patients in the SGLT-2i group and 18,574 patients in the placebo group). Meta-analysis revealed that SGLT-2i treatment significantly reduced all-cause mortality, cardiovascular mortality, and hospitalisation for heart failure (HHF) in patients with cardiovascular disease (all-cause mortality relative risk [RR]: 0.86; 95% confidence interval [CI] 0.81–0.91; *P* < 0.00001; *I*^2^ = 0%; cardiovascular mortality RR: 0.85; 95% CI 0.79–0.92; *P* < 0.0001; *I*^2^ = 26%; HHF RR: 0.69; 95% CI 0.64–0.81; *P* < 0.00001; *I*^2^ = 0%). In patients with HF, mortality and HHF after SGLT-2i treatment for HF with reduced ejection fraction were significantly reduced, whereas HF with preserved ejection fraction did not differ compared with placebo treatment. Moreover, SGLT-2i induced a lower incidence of renal damage and myocardial infarction than the placebo group; however, the risk of infection, amputation, volume depletion, and diabetic ketoacidosis was higher.

**Conclusions:**

SGLT-2i had significant clinical effects on cardiovascular outcomes and significantly influenced acute kidney injury. The effects of SGLT-2i on cardiovascular disease were independent of diabetic status. Sotagliflozin could have advantages over other SGLT-2i in lowering HHF.

**Supplementary Information:**

The online version contains supplementary material available at 10.1186/s12933-021-01272-z.

## Background

The prevalence of cardiovascular disease is increasing, and cardiovascular disease has remained the leading cause of morbidity and mortality worldwide over the last 20 years. Cardiovascular diseases include myocardial infarction, stroke, atherosclerotic cardiovascular diseases, hypertension, atrial fibrillation, and heart failure (HF). Among them, myocardial infarction and stroke are the main causes of complications and death in patients with diabetes mellitus [1]. Among patients with cardiovascular disease, those with type 2 diabetes (T2DM) comprise a higher-risk subgroup, and T2DM has been shown to be an independent risk factor. Long-term diabetes is often complicated by atherosclerosis, HF, and chronic kidney disease. Therefore, for patients with HF or chronic kidney disease combined with diabetes, chances of mortality and hospitalisation for HF (HHF) increase considerably. Many recent clinical studies have shown that sodium-glucose cotransporter type 2 inhibitors (SGLT-2i) have very good clinical effects in patients with T2DM and cardiovascular diseases [2, 3]. Diabetes mellitus is not the only cause of cardiovascular disease, and cardiovascular disease from other causes is associated with a higher risk of poor prognosis. Therefore, any drug needs to be evaluated for efficacy and safety in diverse patients with cardiovascular diseases, regardless of the presence of T2DM as a comorbidity.

SGLT-2i are a new class of medicines for the treatment of T2DM. Inhibiting the expression of SGLT-2 protein in the renal tubules of the kidneys, which reduces the reabsorption of glucose in the kidneys, increases the excretion of glucose in urine and reduces the levels of glucose in the blood plasma [4, 5]. Currently, eight SGLT-2i (canagliflozin, dapagliflozin, empagliflozin, ertugliflozin, luseogliflozin, ipragliflozin, sotagliflozin, and tofogliflozin) have been approved globally for the treatment of T2DM, either as monotherapy or in combination with other hypoglycaemic drugs. In addition, SGLT-2i have been shown to reduce cardiovascular risk events, including lowering blood pressure, improving weight loss, lowering haemoglobin A1c levels, reducing myocardial infarction, reducing stroke, and reducing cardiovascular death in people with T2DM [6, 7]. Over the last 5 years, large randomised controlled trials in patients with T2DM have shown that SGLT-2i improve cardiovascular and renal outcomes, particularly outcomes of patients with HF [8, 9]. AstraZeneca’s dapagliflozin has been approved in the European Union for the treatment of adult patients with symptomatic HF with reduced ejection fraction (HFrEF), regardless of whether the patients have T2DM [10]. However, it is still unclear whether the clinical benefits of SGLT-2i can be extended to all patients with cardiovascular diseases.

Therefore, in the current study, we conducted a meta-analysis of the clinical outcomes of SGLT-2i in patients with cardiovascular diseases, with or without T2DM, and explored the relationships among clinical efficacy, HF subtype, and SGLT-2i type.

## Materials and methods

### Data sources and search strategy

Eligible studies were identified by searching PubMed, EMBASE, Cochrane Library, Web of Science, and ClinicalTrials.gov databases. Searches were conducted by two study investigators independently by the end of November 1, 2020. Search keywords included “cardiovascular disease +”, “CVD”, “cardiovascular death”, “cardiovascular mortality”, “coronary artery disease”, “CAD”, “heart failure”, “HF”, “myocardial infarction”, “stroke”, “+”, “atrial fibrillation”, “SGLT-2”, and “sodium-glucose co-transporter 2 inhibitors”. The search strategies are detailed in Multimedia Additional file 1. The search was restricted to human studies. References for the identified studies were also retrieved to identify studies that may be eligible. Only articles written in English were considered. There were no restrictions with regard to the publication date.

### Inclusion and exclusion criteria

Eligible patients included patients with cardiovascular disease, with or without T2DM. Patients with other age-related comorbidities were not excluded. Patients in the intervention group were treated with SGLT-2i (canagliflozin, dapagliflozin, empagliflozin, ertugliflozin, luseogliflozin, ipragliflozin, sotagliflozin, and tofogliflozin) for a period. Patients who received a placebo or drugs other than SGLT-2i were used as controls. The primary outcomes were all-cause mortality, cardiovascular mortality, and HHF. The secondary outcomes were cardiovascular adverse reactions, endocrine adverse reactions, renal adverse reactions, infection, and other adverse reactions.

### Data extraction and quality assessment

The included studies reported all-cause mortality, cardiovascular mortality, and HHF. All literature titles and abstracts were initially screened for relevance to exclude studies that were not meaningful to our research by two reviewers. Drs. Zheng and Dai independently analysed the full text and extracted data from selected studies; divergence was settled by discussion and consensus or by third-party arbitration. The risk assessment tool for Cochrane bias was used, and the included randomised controlled trials (RCTs) were assessed according to the literature assessment criteria in the Cochrane Systematic Review Manual [11].

### Data synthesis and analysis

Meta-analyses were conducted using Cochrane’s Review Manager (RevMan) version 5.3 (The Cochrane Collaboration, Copenhagen, Denmark) and R programming language, version 3.6.3 (R Foundation for Statistical Computing, Guangzhou, China). We used the associated relative risk (RR) with 95% confidence intervals (CIs) to evaluate clinical outcomes (efficacy and safety) in patients with cardiovascular disease receiving standard treatment with or without SGLT-2i. We assessed whether the results of the included studies were consistent. Chi-square tests were used to assess heterogeneity, and *I*^2^ was used for quantitative analysis. Values of *P* ≥ 0.05 and *I*^2^ ≤ 50% were not considered to represent heterogeneity, and fixed-effect models were used. In contrast, *P* < 0.05 and *I*^2^ > 50% indicated significant heterogeneity among the different studies. Sensitivity analysis was performed by successively deleting each study and reanalysing the dataset for all remaining studies. Publication bias was assessed using funnel plots and Egger’s tests. R software was used to estimate the relevant therapeutic effects and standard errors in the network meta-analysis, and a network meta-model was established to explain the relevant therapeutic effects. The average ranking and surface value under the cumulative ranking curve (SUCRA) of each treatment were calculated. Due to the lack of cycles in research, including the formation of direct and indirect evidence networks, it was impossible to formally assess consistency. *P* values less than 0.05 (two-tailed) were considered statistically significant, and we did not adjust for multiple testing.

## Results

### Search results

**Fig. 1:**
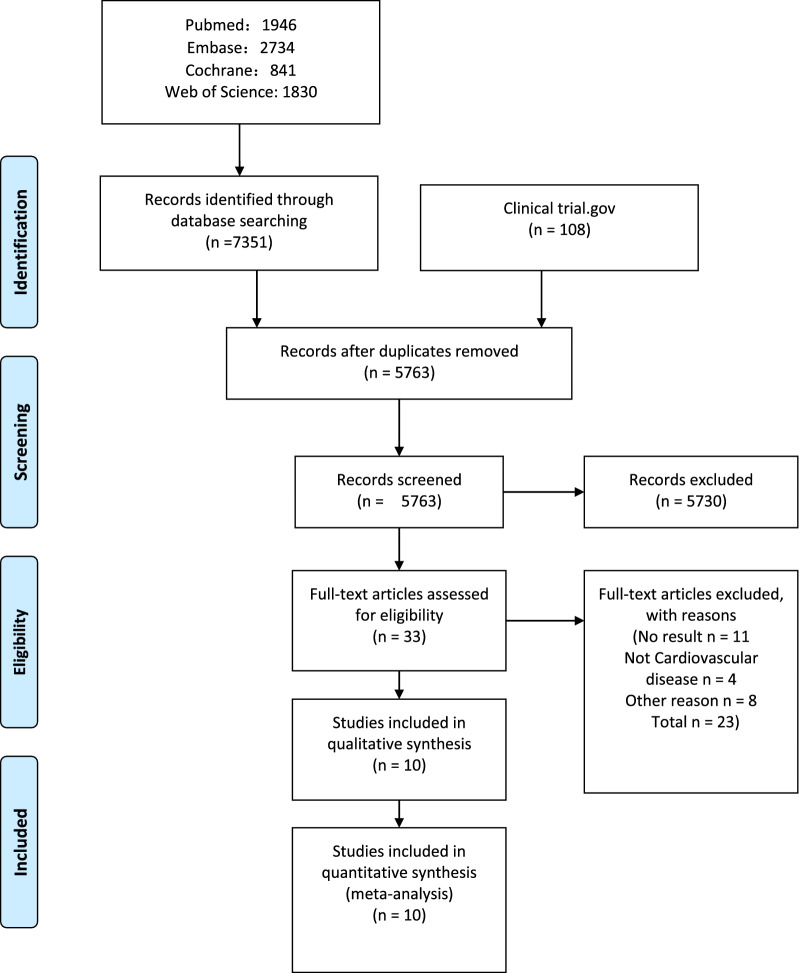
Literature screening and selection flow chart

In total, 7351 articles were retrieved from PubMed, EMBASED, Cochrane Library, and Web of Science databases, and 108 articles were retrieved from ClinicalTrials.gov. After removing duplicate studies, the remaining 5763 articles were screened. After reading 33 eligible full-text articles, 23 were excluded, and 10 studies (total of 43,682 participants) met our inclusion criteria [12–21]. The systematic search results are presented in Fig. 1. HF patient data from cardiovascular outcome trials were available from published post-hoc studies [22, 23].

### Basic characteristics and quality evaluation of the literature

In total, 43,682 patients were included in 10 RCTs (25,108 patients in the intervention group and 18,574 patients in the control group). The detailed baseline characteristics of each study are presented in Additional file 1: Table S1. Quality evaluation was conducted using the Cochrane systematic evaluation method, and the included studies had a low risk of overall bias, as shown in Additional file 1: Figure S1.

### Cardiovascular outcomes

**Fig. 2:**
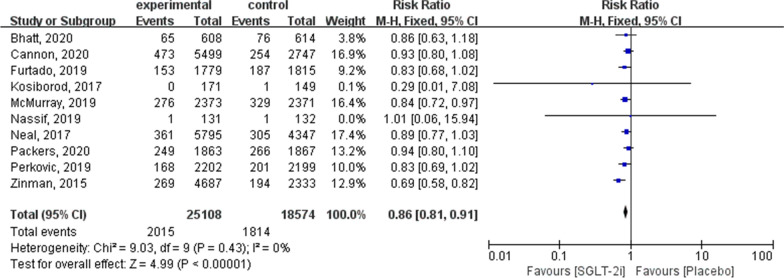
Forest plot of meta-analysis for the comparison of all-cause mortality between the SGLT-2i and placebo groups

**Fig. 3:**
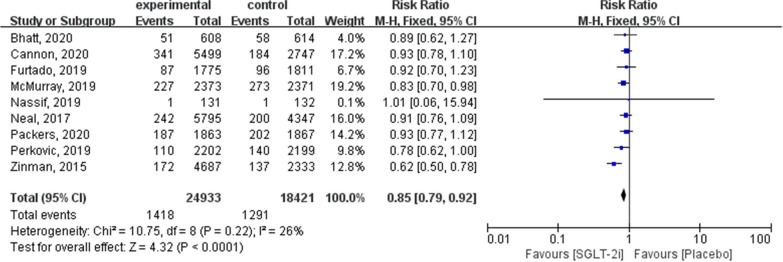
Forest plot of meta-analysis for the comparison of cardiovascular mortality between the SGLT-2i and placebo groups

**Fig. 4:**
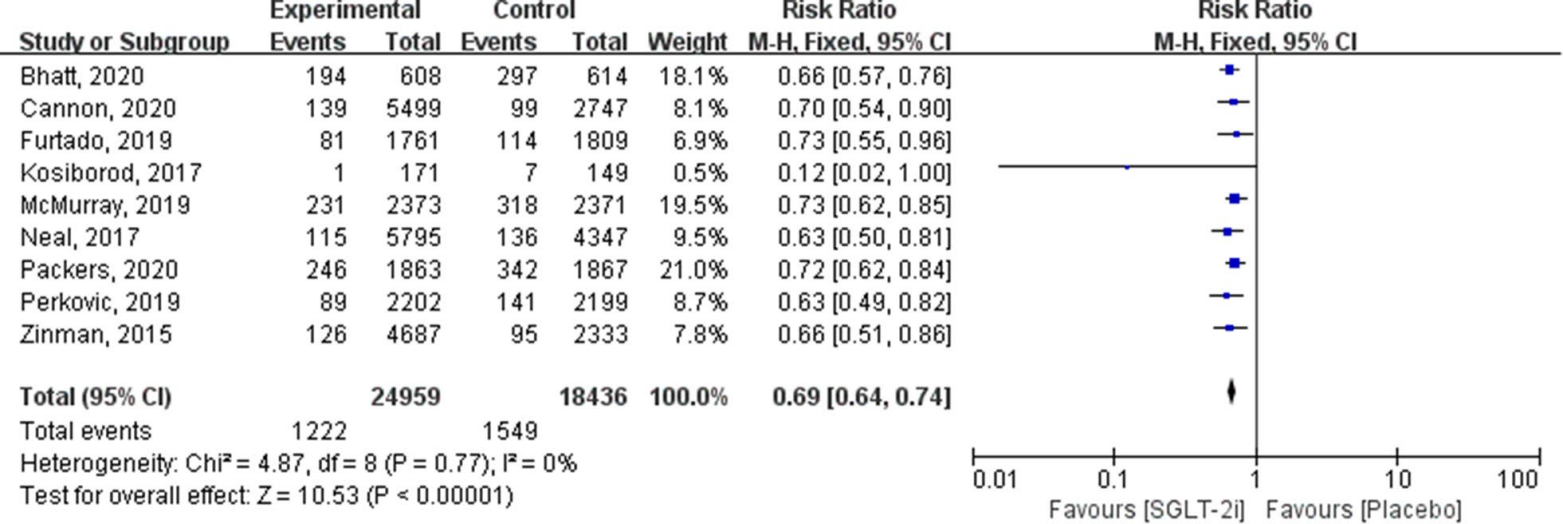
Forest plot of meta-analysis for the comparison of HHF between the SGLT-2i and placebo groups

**Fig. 5:**
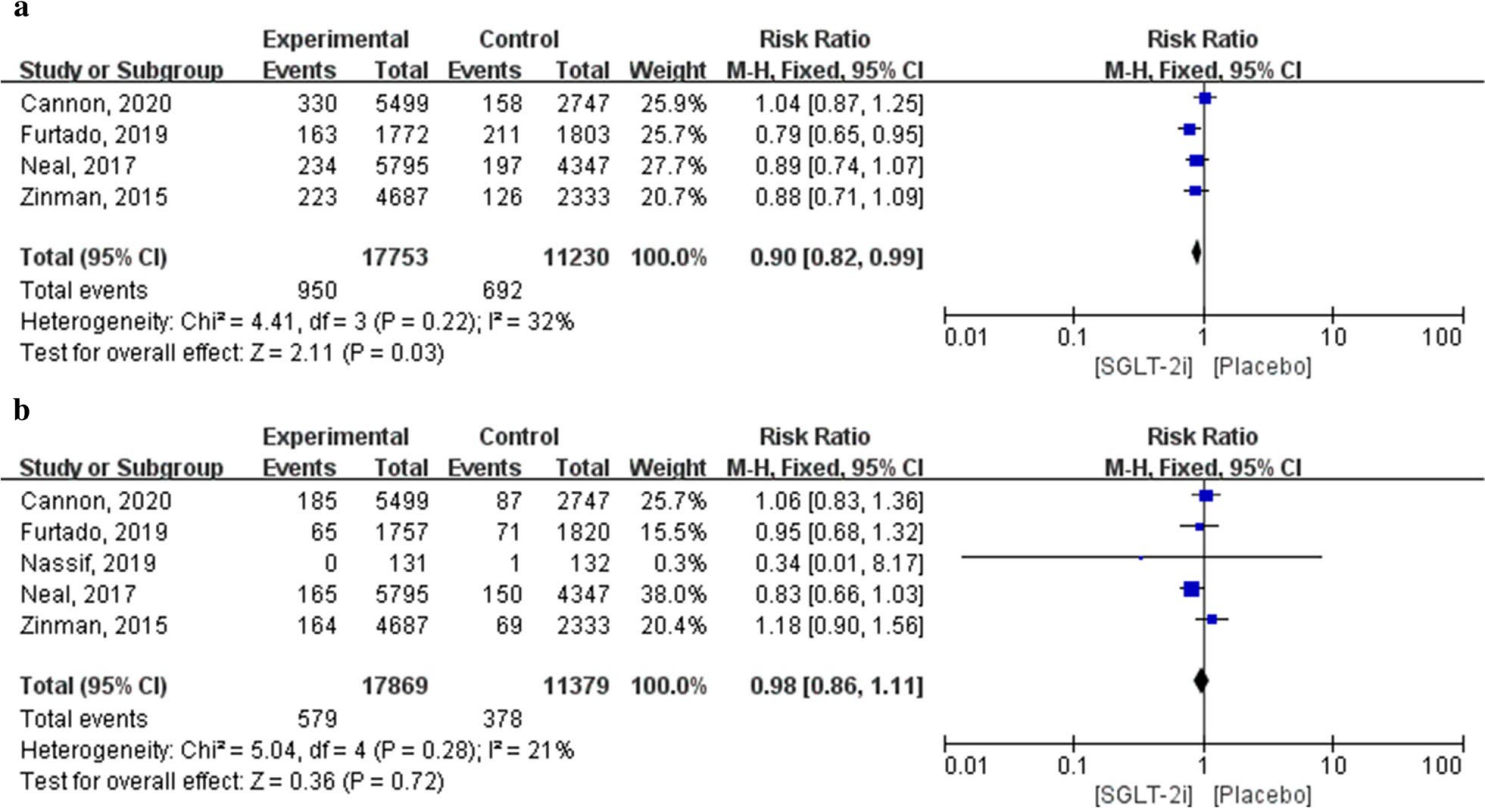
Forest plot of meta-analysis for the comparison of adverse cardiovascular events between SGLT-2i and placebo groups. **a** Myocardial infarction adverse outcomes, **b** stroke adverse outcomes

The summarised outcomes of our meta-analysis are presented in Additional file 1: Table S2. To evaluate the primary outcomes, 10 trials were included in the meta-analysis. Estimates of cardiovascular outcomes for all-cause mortality, cardiovascular mortality, HHF, and adverse cardiovascular outcomes are shown in Figs. 2, 3, 4 and 5.

#### All‑cause mortality

All 10 articles contained all-cause mortality outcomes, with a total of 43,682 patients (25,108 in the SGLT-2i group and 18,574 in the placebo group; RR: 0.86; 95% CI 0.81–0.91; *P* < 0.00001; *I*^2^ = 0 %; Fig. 2).

#### Cardiovascular mortality

Nine articles contained outcomes of cardiovascular death (24,933 in the SGLT-2i group and 18,421 in the placebo group). Subgroup analysis revealed that cardiovascular death was significantly lower in patients using SGLT-2i than in the placebo group (RR: 0.85; 95% CI 0.79–0.92; *P* < 0.0001; *I*^2^ = 26 %; Fig. 3).

#### HHF

Nine articles included HHF outcomes (24,959 in the SGLT-2i group and 18,436 in the placebo group). Meta-analysis results showed that HHF was significantly lower in patients using SGLT-2i than in the placebo group (RR: 0.69; 95% CI 0.64–0.74; *P* < 0.00001; *I*^2^ = 0 %; Fig. 4).

#### Adverse cardiovascular outcomes

Five studies reported adverse cardiovascular events, including four with myocardial infarction. The five studies included 28,983 patients (17,753 in the SGLT-2i group and 11,230 in the placebo group). Meta-analysis results showed that the incidence of myocardial infarction was significantly lower in patients receiving SGLT-2i than in those receiving a placebo (RR: 0.90; 95% CI 0.82–0.99; *P* = 0.03; *I*^2^ = 32%). Five studies with stroke outcomes included 29,248 patients (17,869 in the SGLT-2i group and 11,379 in the placebo group). Meta-analysis results indicated that there were no significant differences in the incidence of stroke between patients treated with SGLT-2i and those treated with a placebo (RR: 0.98; 95% CI 0.86–1.11; *P* = 0.72; *I*^2^ = 21%; Fig. 5).

### Renal outcomes

#### Renal composite outcomes

**Fig. 6:**
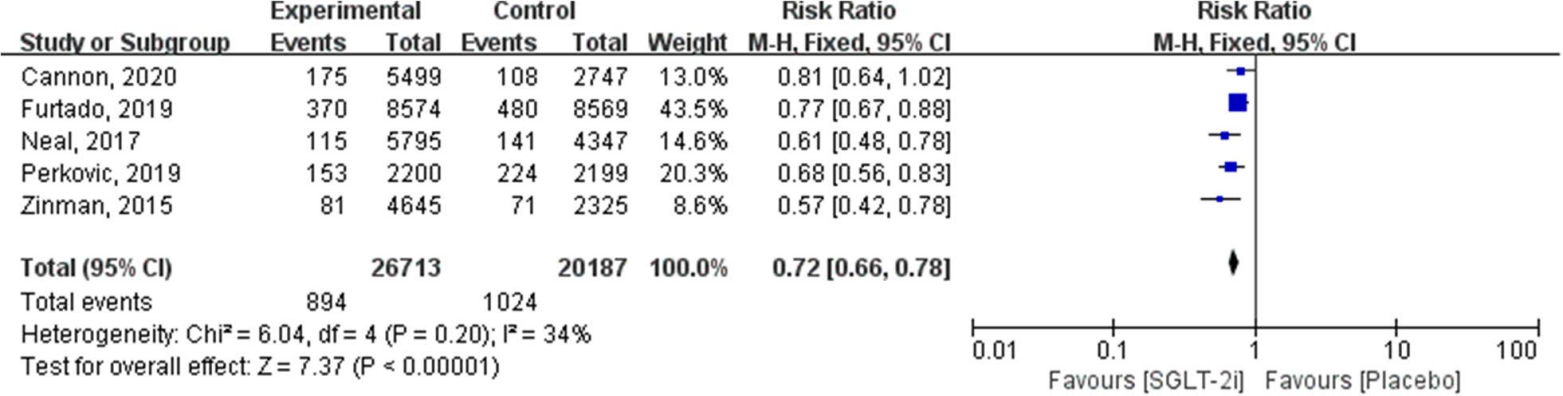
Forest plot of meta-analysis for the comparison of renal composite outcomes between SGLT-2i and placebo groups

Based on five trials, including a total of 46,900 patients, SGLT-2i significantly reduced the risk of the renal composite outcomes (RR: 0.72; 95% CI 0.66–0.78; *P* < 0.00001; *I*^2^ = 34 %; Fig. 6).

#### Adverse renal outcomes

**Fig. 7:**
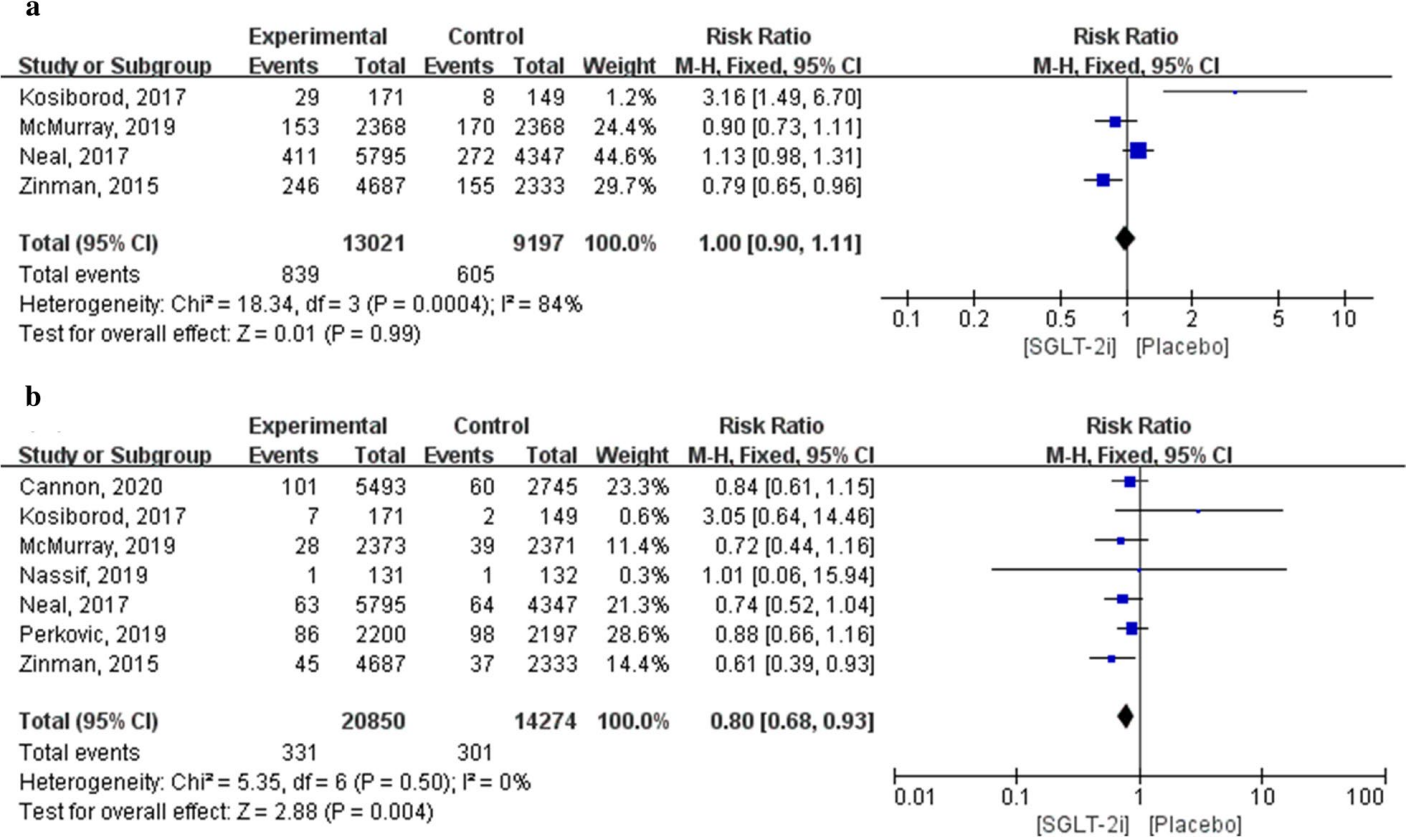
Forest plot of meta-analysis for the comparison of adverse renal events between the SGLT-2i and placebo groups. **a** Acute renal failure, **b** acute renal injury

In the four trials (EMPA-REG OUTCOME, CANVAS, DAPA-T2DM and HF, and DAPA-HF), there were no significant differences in compound outcomes of acute renal failure between the SGLT-2i and placebo groups (RR: 1.00; 95% CI 0.90–1.11; *P* = 0.99; *I*^2^ = 84 %). Sensitivity analysis was performed by sequentially deleting each study and reanalysing the datasets for all remaining studies (RR: 0.99, 95% CI 0.90–1.10, *P* = 0.96; *I*^2^ = 80.8%; Additional file 1: Table S3). In total, 35,124 patients with acute renal injury were included in seven articles (20,850 in the SGLT-2i group and 14,274 in the placebo group). Meta-analysis results showed that the incidence of acute renal injury in patients treated with SGLT-2i was significantly lower than that in patients treated with a placebo (RR: 0.80; 95% CI 0.68–0.93; *P* = 0.004; *I*^2^ = 0%; Fig. 7).

### Safety outcomes

#### Adverse endocrine outcomes

In total, 30,719 patients (18,645 in the SGLT-2i group and 12,074 in the placebo group) were included in the six studies on hypoglycaemia outcomes. Meta-analysis results showed that there were no significant differences in the incidence of hypoglycaemia between patients using SGLT-2i and those using the placebo (RR: 1.00; 95% CI 0.96–1.05; *P* = 0.87; *I*^2^ = 8%).

**Fig. 8:**
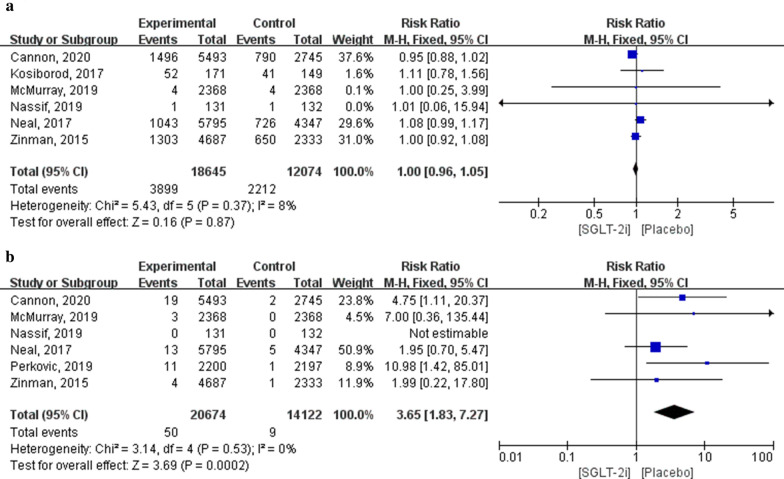
Forest plot of meta-analysis for the comparison of adverse endocrine events between the SGLT-2i and placebo groups. **a** Hypoglycaemia adverse outcomes, **b** diabetic ketoacidosis adverse outcomes

Additionally, 34,796 patients (20,674 in the SGLT-2i group and 14,122 in the placebo group) were included in six studies of outcomes related to diabetic ketoacidosis. Meta-analysis results showed that the incidence of diabetic ketoacidosis in patients treated with SGLT-2i was significantly higher than that in patients treated with a placebo (RR: 3.65; 95% CI 1.83–7.27; *P* = 0.0002; *I*^2^ = 0%; Fig. 8).

#### Adverse infection outcomes

Outcomes of infection, including urinary tract infection, male genital infection, and female genital infection, were evaluated. The results suggested that the incidence of infection in patients treated with SGLT-2i was significantly higher than that in patients treated with a placebo (*P* < 0.05). The results of urinary tract infection were assessed in four articles, which included a total of 25,720 patients (16,146 in the SGLT-2i group and 9574 in the placebo group). Meta-analysis results showed that the incidence of urinary tract infection in patients treated with SGLT-2i was significantly higher than that in patients treated with a placebo (RR: 1.08; 95% CI 1.01–1.15; *P* = 0.02; *I*^2^ = 39%).

**Fig. 9:**
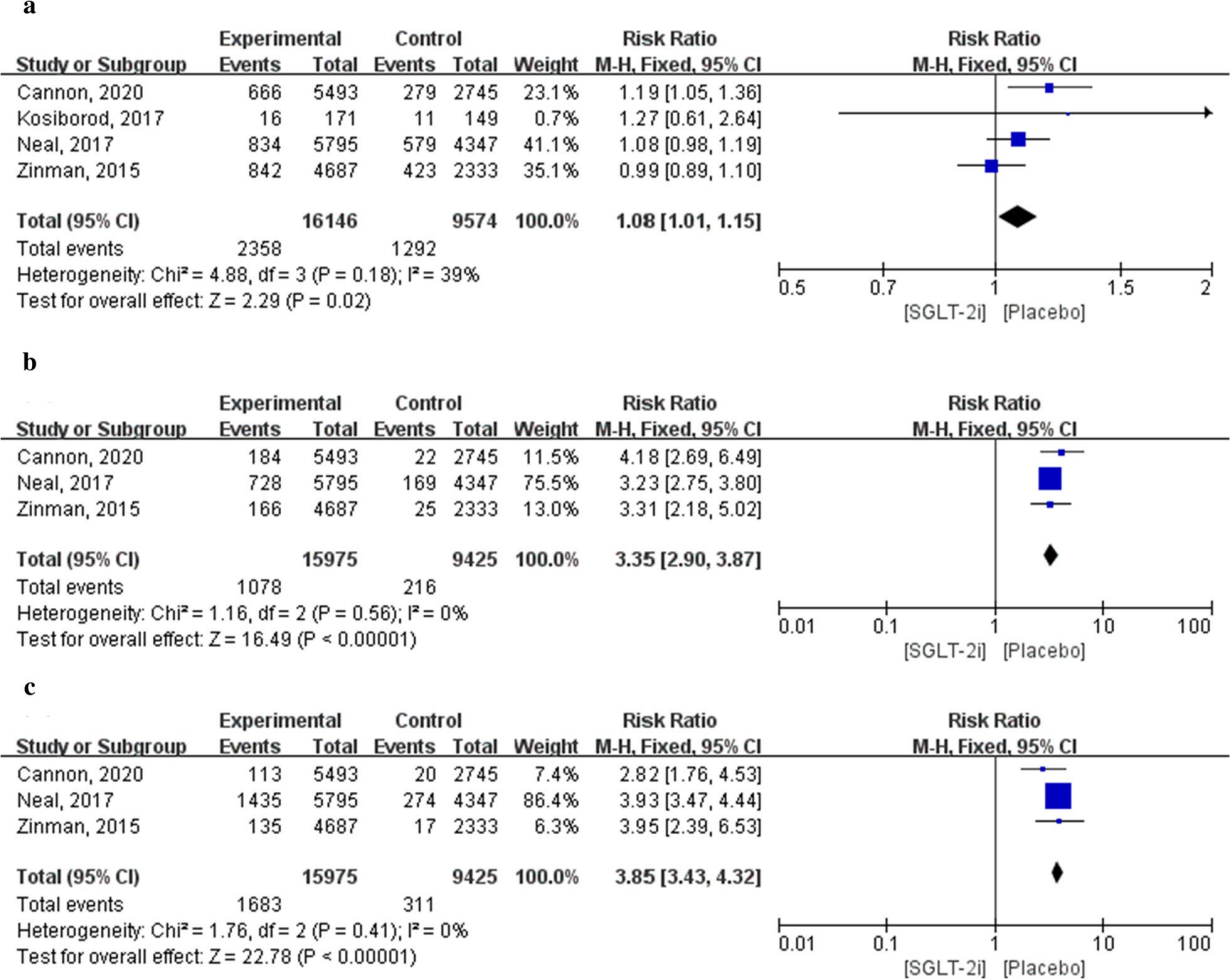
Forest plot of meta-analysis for the comparison of infection adverse events between the SGLT-2i and placebo groups. **a** Urinary tract infection adverse outcomes, **b** male genital infection adverse outcomes, **c** female genital infection adverse outcomes

Results of male and female genital infections were included in the three articles, which involved a total of 25,400 patients (15,975 in the SGLT-2i group and 9425 in the placebo group). Meta-analysis results showed that the incidence of male and female genital infections in patients treated with SGLT-2i was significantly higher than that in patients treated with a placebo (male: RR: 3.35; 95% CI 2.90–3.87; *P* < 0.00001; *I*^2^ = 0%; female: RR: 3.85; 95% CI 3.43–4.32; *P* < 0.00001; *I*^2^ = 0%; Fig. 9).

#### Others

Five articles containing fracture outcomes included 34,533 patients (20,543 in the SGLT-2i group and 13,990 in the placebo group). Meta-analysis results showed that there were no significant differences in fracture incidence between patients treated with SGLT-2i and those treated with a placebo (RR: 1.11; 95% CI 0.99–1.23; *P* = 0.08; *I*^2^ = 24%).

Four articles included outcomes of amputation involving 27,513 patients (15,856 in the SGLT-2i group and 11,657 in the placebo group). Meta-analysis results showed that the incidence of amputation in patients treated with SGLT-2i was significantly higher than that in patients treated with a placebo (RR: 1.42; 95% CI 1.18–1.71; *P* = 0.0002; *I*^2^ = 32%).

**Fig. 10:**
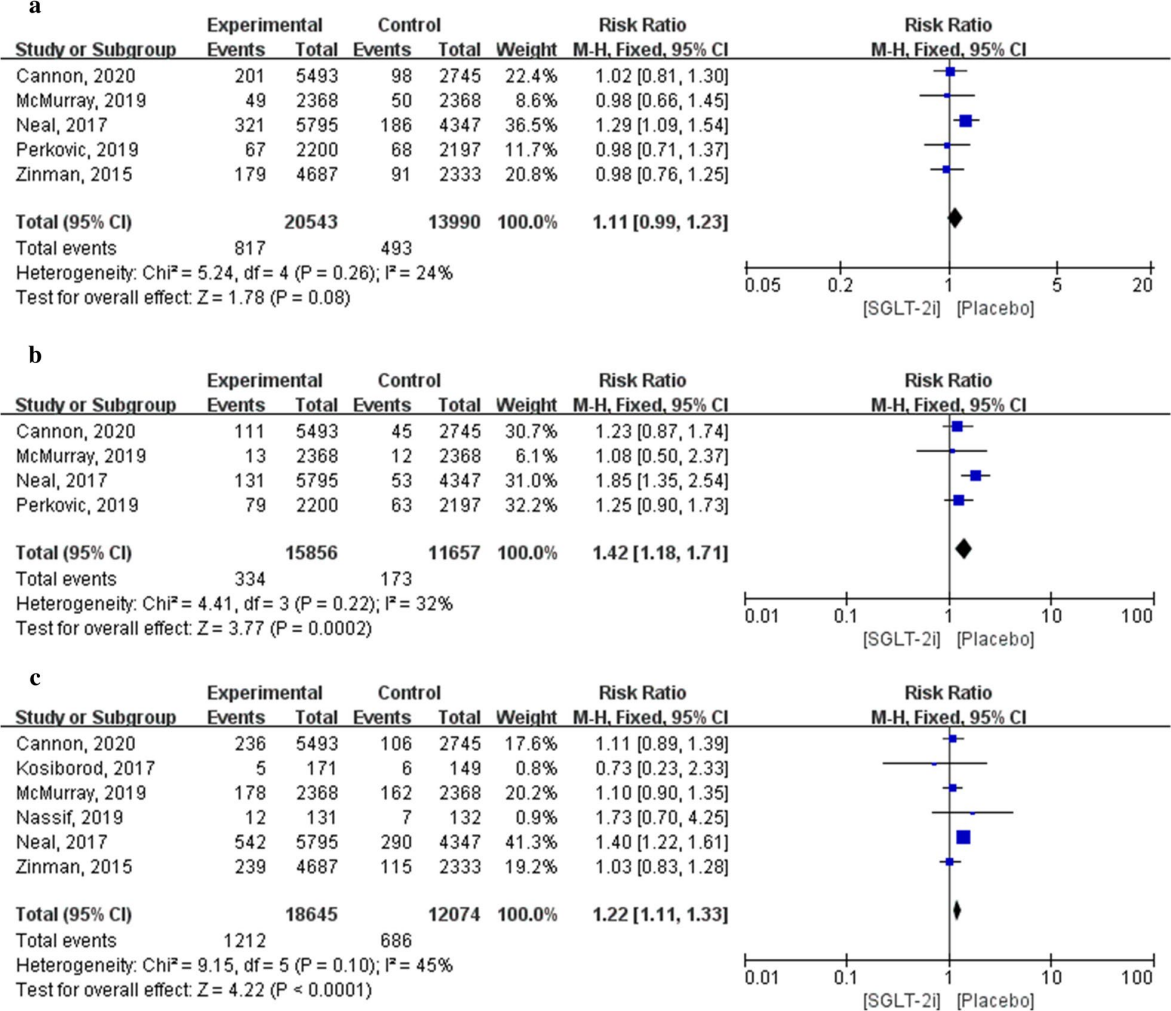
Forest plot of meta-analysis for the comparison of other adverse events between the SGLT-2i and placebo groups. **a** Fracture adverse outcomes, **b** amputation adverse outcomes, **c** volume depletion adverse outcomes

Six articles included outcomes of volume depletion in 30,719 patients (18,645 in the SGLT-2i group and 12,074 in the placebo group). Meta-analysis results showed that the volume depletion of patients treated with a placebo was significantly lower than that of patients treated with SGLT-2i (RR: 1.22; 95% CI 1.11–1.33; *P* < 0.0001; *I*^2^ = 45%; Fig. 10).

### Subgroup analysis

Detailed forest plots displaying subgroup analyses are presented in Additional file 1. For all outcomes in the overall cardiovascular disease population, no subgroup effect was observed upon stratification by T2DM status (Additional file 1: Figs. S2–S4). In a subgroup analysis of HF types, there were no significant differences in all-cause mortality, cardiovascular mortality, or HHF between the SGLT-2i and placebo groups among patients with HF with preserved ejection fraction (HFpEF; Additional file 1: Figs. S5–S7). SGLT-2i reduced all-cause mortality, cardiovascular mortality, and HHF largely independent of drug type, with the exception of ertugliflozin and sotagliflozin (Additional file 1: Figs. S8–S10). In terms of safety, only the subgroup analysis of ketoacidosis and acute renal injury was performed because of the limited number of included trials. In terms of ketoacidosis, canagliflozin and ertugliflozin showed significant differences compared with the placebo group, whereas the other drugs showed no significant differences (Additional file 1: Fig. S11). There were no significant differences in acute renal injury between patients administered dapagliflozin, canagliflozin, or ertugliflozin and patients administered a placebo. Empagliflozin significantly reduced the number of acute renal injury events (Additional file 1: Fig. S12).

### Bias analyses and numerical network meta‐analysis

**Fig. 11:**
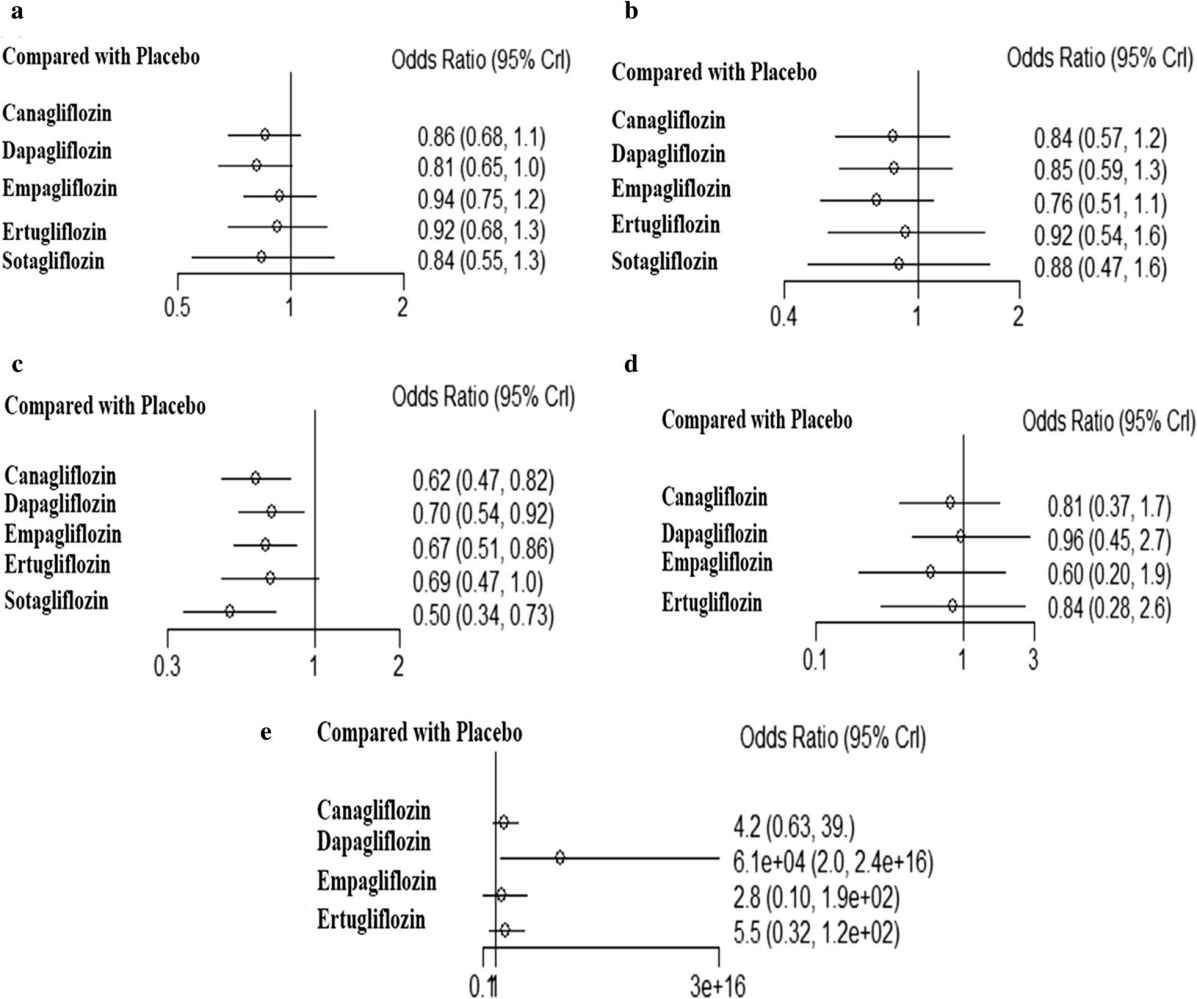
Network meta-analysis for all outcomes of each drug compared with placebo. **a** All-cause mortality, **b** cardiovascular mortality, **c** HHF, **d** acute renal injury, **e** diabetic ketoacidosis adverse outcomes

A network meta-analysis was performed for all outcomes for each drug, as shown in Fig. 11. According to the SUCRA results, the rankings of the efficacy of the six drugs and placebo are shown in Additional file 1: Table S4. Dapagliflozin reduced all-cause mortality to the greatest extent, but had the highest risk of ketoacidosis. Sotagliflozin significantly reduced HHF. There were no significant differences in the effects of SGLT-2i on other outcomes.

### Sensitivity analysis results

**Fig. 12:**
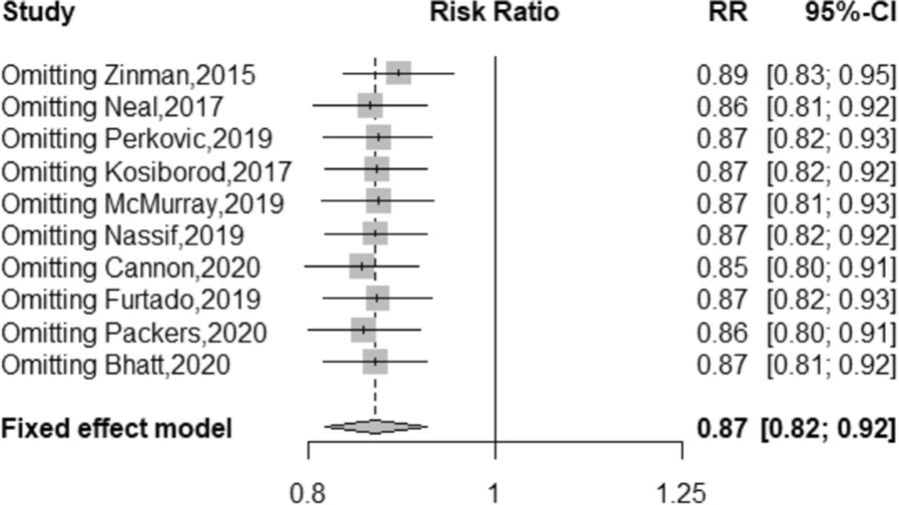
Forest plot of sensitivity analysis for all-cause mortality excluding individual studies

Sensitivity analysis was performed to compare all-cause mortality by sequentially removing one study at a time and observing the exaggerated effect this had on the overall results. As shown in Fig. 12, after each study was excluded, the pooled RR of the remaining included studies was approximately 0.87, with no significant change, suggesting that the results of this meta-analysis were stable and reliable. Removing any study did not affect the overall results.

### Publication bias

**Fig. 13:**
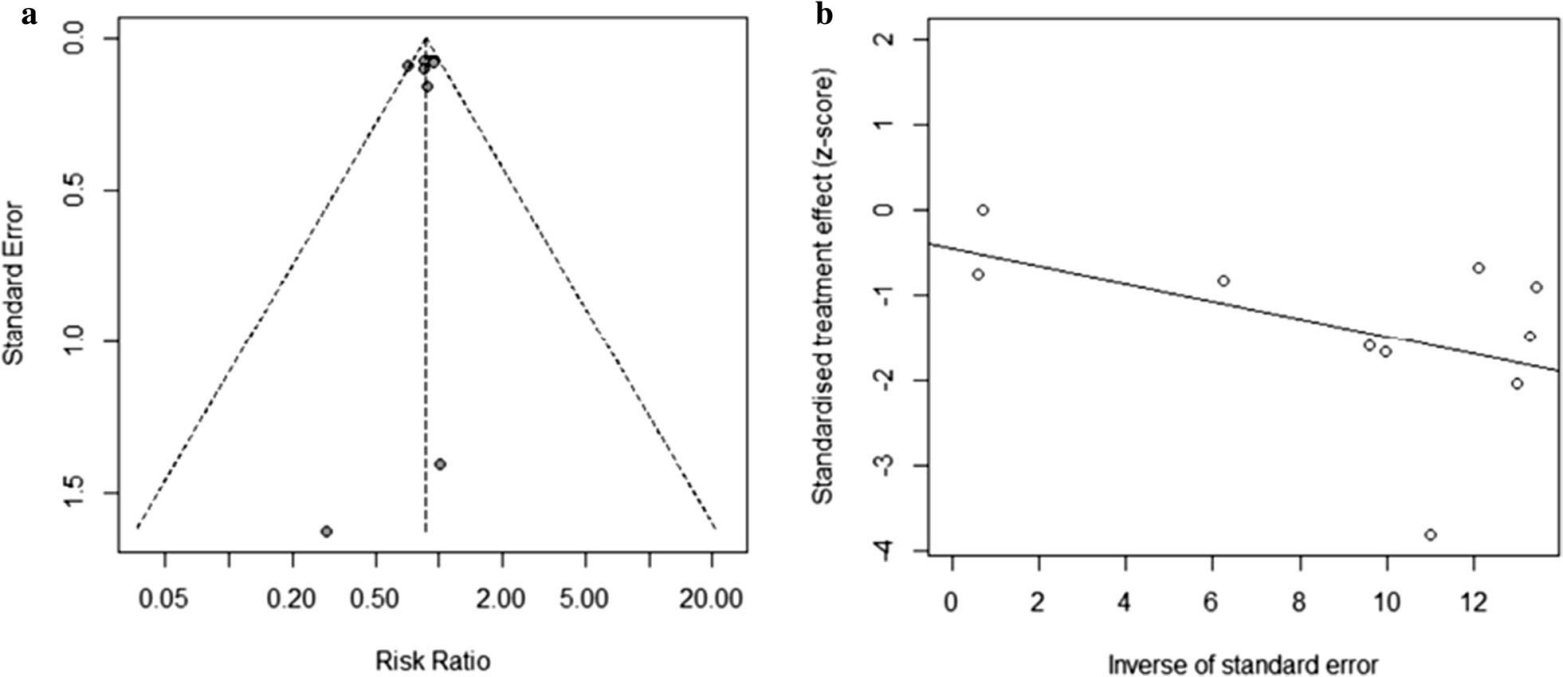
**a** Funnel plot of all-cause mortality. **b** Egger’s funnel plot of all-cause mortality

Using funnel plots drawn by Review Manager 5.3, we visually assessed the publication bias of all-cause mortality (Egger’s test *P* = 0.3638, 95% CI – 0.662 to 0.669). All evidence suggested that the probability of publication bias was low. Funnel plots were not generated for other comparisons because fewer than 10 studies were included (Fig. 13).

## Discussion

To the best of our knowledge, this is the first study comparing SGLT-2i with a placebo in comprehensive outcomes of primary efficacy and adverse events in cardiovascular diseases in patients with and without T2DM. Cardiovascular diseases include various conditions, such as common coronary artery disease, hypertension, dyslipidaemia, congenital heart disease, valvular disease, myocardial infarction, stroke, and arrhythmia. Accordingly, cardiovascular diseases are a serious threat to human health worldwide. Cardiovascular outcomes, survival, and prognosis in patients with cardiovascular diseases are relatively poor. In a previous meta-analysis, SGLT-2i treatment of patients with T2DM significantly reduced all-cause mortality, cardiovascular mortality, HHF, risk of HF, and renal failure [24–28]. Moreover, the main findings of this meta-analysis demonstrated that in patients with cardiovascular disease, regardless of the presence or absence of T2DM, SGLT-2i significantly reduced mortality and major adverse cardiovascular events (MACEs) compared with the placebo, and the benefit for the prevention and treatment of HF and renal disease was consistent with that of earlier meta-analyses [29]. Similar results were observed in the HFrEF population; however, there were no significant differences in patients with HFpEF. Furthermore, no significant differences were observed between the two groups for most adverse events. The results for the risk of infection, amputation, and hypovolemia should be interpreted with caution. Dapagliflozin significantly reduced all-cause mortality, but its risk of ketoacidosis was the highest, whereas sotagliflozin significantly reduced HHF.

SGLT-2i, due to their specific mechanism of blocking the sodium-glucose cotransporter, offer some benefits that may be translated into cardiovascular outcomes. For example, these compounds have been reported to lower blood pressure [30], blood lipid levels [31, 32], and body weight [32, 33]; however, the exact mechanisms through which such drugs improve cardiovascular outcomes remain unclear. In a systematic review and meta-analysis based on 43 randomized controlled studies, it was demonstrated that SGLT-2i were not associated with heart rate changes [34]. The 12-week long EMPA-REG BP trial including 823 participants with T2DM demonstrated that the blood pressure benefit of SGLT-2i was obvious, regardless of the number and type of antihypertensive drugs used by the participants at baseline. Two small, open-label, single-site studies with Japanese cohorts have suggested that use of SGLT-2i is associated with reductions in the levels of atherogenic, small, dense low-density lipoprotein cholesterol [35].

In the EMPA-REG OUTCOME trial, all eligible patients had established cardiovascular disease, and empagliflozin reduced the risk of all-cause mortality by 32%, cardiovascular mortality by 38%, and HHF by 35% compared with the placebo. At the same time, the study confirmed that the dose of empagliflozin did not affect the hazard ratios of cardiovascular outcomes. In the CANVAS and CANVAS-R trials, treatment resulted in reductions of 49% for all-cause mortality, 47% for cardiovascular mortality, 30% for HHF, and 22% for major adverse cardiovascular events. Patients in the canagliflozin group had a lower risk of cardiovascular mortality, HHF, and renal failure than those in the placebo group at a median follow-up of 2.62 years in the CREDENCE trial. In the DECLARE-TIMI 58 trial, dapagliflozin appeared to robustly reduce the risk of cardiovascular mortality or HHF and MACEs in patients with T2DM and previous myocardial infarction. In the VERTIS-CV trial involving patients with T2DM and established atherosclerotic cardiovascular disease, ertugliflozin was shown to be noninferior to the placebo with MACEs. However, the incidence of cardiovascular mortality or HHF did not differ significantly between the two groups. Owing to the diverse properties of different drugs, we cannot exclude the possibility that differences among the agents in this class may result in real differences in clinical outcomes. It is also possible that the effects of individuals are similar because the confidence interval of VERTIS CV overlaps with that of previous trials. The main mechanisms of the cardiovascular benefits of SGLT-2i include reduced plasma volume and diuresis, reduced ventricular remodelling, myocardial metabolism, and adipokine dynamics. However, further basic research is required to elucidate the specific pathophysiological mechanisms underlying the cardiovascular benefits of SGLT-2i.

Renal damage not only has metabolic adverse outcomes but also increases the risk of renal and cardiovascular morbidity and mortality. Initially, the inhibitory effects of SGLT-2i can lead to tubule-glomerular negative feedback by enhancing natriuresis and sodium delivery to dense plaques, thereby reducing diabetic glomerular hyperfiltration caused by hyperglycaemia. SGLT-2i are thought to reduce chronic renal damage caused by hyperglycaemia [36]. The significant increase in haematocrit concentrations in patients receiving SGLT-2i appears to be associated with increased production of erythropoietin, which may also increase oxygen delivery to the kidneys and reduce renal hypoxia. In addition to their effects on haemodynamics, SGLT-2i have beneficial metabolic effects that may facilitate renal protection. Our results were consistent with the findings of previous meta-studies and showed that SGLT-2i could significantly reduce further deterioration of renal function [29, 37].

There is sufficient evidence that SGLT-2i may be more effective in diabetic kidney disease than in non-diabetic nephropathy, and physicians are encouraged to initiate SGLT-2i treatment in diabetic patients as early as possible in order to delay the progression of renal complications of diabetes [38]. Meta-analysis of three major clinical trials (EMPA-REG OUTCOME, CANVAS Program, and DECLARE-TIMI 58 Trial) showed that SGLT-2i could significantly reduce the incidence of adverse event endpoints in patients compared with placebo (eGFR continued to decrease by more than 40%, developed to end-stage renal disease and renal death) and improve outcomes of renal disease (decreased by 43%). Such protective effects were consistent for patients with different eGFR baseline levels. Therefore, the renal benefits of SGLT-2i administration were obvious.

In terms of safety, three available studies showed neutral effects on myocardial infarction and stroke in patients receiving SGLT-2i [39–41]. In an RCT and a nationwide cohort study, the results showed no increased risk of hypoglycaemia with SGLT-2i monotherapy [42, 43]; however, SGLT-2i were associated with approximately twice the risk of diabetic ketoacidosis as DPP4 inhibitors [44]. Accordingly, clinicians should be cautious when combining SGLT-2i with other hypoglycaemic drugs. SGLT-2i reduced the risk of dialysis, transplantation, or death due to kidney disease in individuals with T2DM, with or without basic renal disease, and provided protection against acute kidney injury [45–47]. Currently, common adverse reactions to SGLT-2i observed in the clinical setting including fungal infection of the genital system, urinary system infection, hypovolemia, ketoacidosis, fracture, and amputation.

The use of SGLT-2i was not associated with an increased risk of fracture and amputation compared with other antidiabetics [44, 48–50]. Previous meta-analyses have shown that SGLT-2i do not increase the risk of fracture or amputation; however, the heterogeneity of these studies was high [51].Volume depletion is another major concern, not consistent with recent studies, which showed significant differences between SGLT-2i and other oral hypoglycaemic agents in volume depletion events [52–54], perhaps because of individualised dosing of SGLT-2i. In a cardiovascular outcome trial of SGLT-2i, the most common adverse event was genitourinary infection, which may be attributed to the selective inhibition of renal proximal tubule glucose reabsorption by SGLT-2i. According to the United States Food and Drug Administration, all manufacturers are required to add a warning of potential infection to the prescribing information and patient medication guide for all SGLT-2i in 2018. Although these adverse events should not mask the overall cardio-renal benefits of SGLT-2i [55–57], individuals at risk of these complications should be monitored closely, and treatment should be reconsidered or discontinued if such complications occur.

In a meta-analysis on a HFrEF population, including 8474 patients in two trials (EMPEROR-Reduced [58] and DAPA-HF [17]), researchers noted a 13% reduction in all-cause mortality and a 14% reduction in cardiovascular mortality. Meta-analysis of the diabetic status subgroup showed that SGLT-2i significantly reduced all-cause and cardiovascular mortality, HF hospitalization, and severe adverse renal outcomes, and no statistical evidence of the heterogeneity of the treatment effects for any of these endpoints was obtained [59]. Previous studies have shown that SGLT-2i have favourable effects on cardiovascular risk in patients with T2DM, and our study found that SGLT-2i also have significant cardiovascular and renal benefits in patients with cardiovascular disease without T2DM. The heterogeneity of the associations with outcomes of different SGLT2i on cardiovascular, renal, and safety outcomes among patients with T2DM and CVD should be evaluated in further studies.

In current research on HFpEF, only the subgroup analyses in the DECLARE-TIMI 58 and VERTIS-CV trials suggested a potential impact on HHF or cardiovascular mortality. SGLT-2i may prevent HF because, in addition to its glucose-lowering effects, SGLT-2i also has a number of mechanisms unrelated to glucose-lowering, such as reducing preload and afterload through natriuretic and osmotic diuresis, improving cardiac metabolism and bioenergy, reducing myocardial necrosis and fibrosis, and adipokine changes. A retrospective study found that SGLT-2i were associated with improvements in left ventricular geometry and systolic and diastolic function in patients of T2DM. Compared with patients without HF, SGLT-2i improved cardiac function to a greater extent. Moreover, in diabetic patients with HF, SGLT-2i may exert volume reduction effects, which would be more beneficial in HFrEF; however, the benefits would be blunted in HFpEF [60]. Two ongoing clinical trials in patients with HFpEF will provide further evidence of its efficacy (NCT03057951 and NCT01297257).

Among SGLT-2i, sotagliflozin is the first dual target inhibitor of SGLT-1/2 to inhibit both sodium-glucose co-transporter 1 and sodium-glucose co-transporter 2. SGLT-2 inhibition increases urinary glucose excretion, whereas SGLT-1 inhibition reduces postprandial glucose levels by delaying intestinal glucose absorption. In patients with type 2 diabetes with decompensated episodes of HF, initiation of sotagliflozin treatment before or shortly after discharge reduces the incidence of cardiovascular death and hospitalization or emergency visits for HF. SGLT-1 is mainly located at the brush margin of the small intestinal mucosa and at the S3 segment of the proximal concocted tubules of the kidney, and SGLT-2 is mainly located at the S1 segment of the proximal concocted tubules of the kidney. Importantly, 90% of the glucose in the glomerular filtration fluid is absorbed by SGLT-2, and only 10% is absorbed by SGLT-1 [61, 62]. Adverse events such as hypotension, hypoglycaemia, and diarrhoea are common in patients administered sotagliflozin, which may be related to its effects on SGLT-1 receptor. Overall, each of the SGLT-2 inhibitors showed similar trends of improvement in different cardiovascular outcomes. Although the small differences in effect size could be explained by trial-based heterogeneity and slight discrepancies in patient comorbidities, these results need to be further explored in future studies. Compared with other hypoglycaemic agents, a recent network meta-analysis suggested that SGLT-2i and GLP-1 receptor agonists, when added to other hypoglycaemic regimens, significantly reduce mortality, non-fatal myocardial infarction, renal failure, and severe hyperglycaemia [63]. Compared with GLP-1 agonists, SGLT-2i reduced all-cause mortality and the number of HHF, whereas GLP-1 agonists only reduced non-fatal strokes more than SGLT-2i [64].

Our study had some limitations. First, confounding factors, such as age, sex, regionalism, baseline haemoglobin A1c, estimated glomerular filtration rate, and exposure to cardiovascular disease-related drugs, as well as drug combinations and other potential factors, were difficult to control. Second, articles published in languages other than English were excluded. Third, few RCTs of certain SGLT-2i in the HFpEF population have been published. Finally, this meta-analysis may be underpowered for comparison of long-term adverse events between SGLT-2i and the placebo owing to the different durations of follow up for the included RCTs. Therefore, additional studies are required to confirm our findings.

## Conclusions

In conclusion, according to the results of the current study, SGLT-2i can effectively reduce major cardiovascular events and renal events in patients with cardiovascular disease, and the impact of these drugs did not seem to be influenced by T2DM. However, there were no significant effects in patients with HFpEF. Moreover, there was no clear evidence of additional safety concerns over SGLT-2i use in the treatment of cardiovascular disease. However, the robustness of the findings should be further confirmed in dedicated cardiovascular outcome trials.

## Supplementary Information

**Additional file 1:** **1.** Search algorithm. **2. Table S1.** Characteristics of studies included in the systematic review. **3.** **Figure S1.** Evaluation of randomized controlled trials. **4.** **Table S2.** Results of meta-analysis comparison of SGLT-2i inhibitors and placebo. **5.** **Figure S2.** The primary outcome of SGLT-2i and placebo on the all-cause mortality in T2DM subgroup. **6.** **Figure S3.** The primary outcome of SGLT-2i and placebo on the Cardiovascular mortality in T2DM subgroup. **7.** **Figure S4.** The primary outcome of SGLT-2i and placebo on the HHF in T2DM subgroup. **8.** **Figure S5.** The primary outcome of SGLT-2i and placebo on the all-cause mortality in heart failure subgroup. **9.** **Figure S6.** The primary outcome of SGLT-2i and placebo on the Cardiovascular mortality in heart failure subgroup. **10.** **Figure S7.** The primary outcome of SGLT-2i and placebo on the HHF in heart failure subgroup. **11.** **Figure S8.** The primary outcome of SGLT-2i and placebo on the all-cause mortality in individual drugs subgroup. **12.** **Figure S9.** The primary outcome of SGLT-2i and placebo on the Cardiovascular mortality in individual drugs subgroup. **13.** **Figure S10.** The primary outcome of SGLT-2i and placebo on the HHF in individual drugs subgroup. **14.** **Figure S11.** The adverse reactions of SGLT-2i and placebo on the ketoacidosis in individual drugs subgroup. **15.** **Table S3.** Sensitivity analysis of acute renal failure. **16.** **Figure S12.** The adverse reactions of SGLT-2i and placebo on the acute renal injury in individual drugs subgroup. **17.** **Table S4.** Rankogram of the individual drugs.

## Data Availability

Not applicable.

## Abbreviations

T2DM: type 2 diabetes
HHF: Hospitalisation for heart failure
SGLT-2i: Sodium-glucose cotransporter type 2 inhibitors
HFrEF: Heart failure with reduced ejection fraction
HFpEF: Heart failure with preserved ejection fraction
RCT: Randomised controlled trial
RR: Relative risk
MACE: Major adverse cardiovascular event
