# Supplementary material for: Effects of sodium‐glucose cotransporter type 2 inhibitors on cardiovascular, renal, and safety outcomes in patients with cardiovascular disease: a meta‐analysis of randomized controlled trials
Source: Cardiovasc Diabetol. 2021 Apr 22;20:83. doi: 10.1186/s12933-021-01272-z (PMC8063441; doi:10.1186/s12933-021-01272-z)
Supplement: Supplementary file 1 — Additional file 1: 1. Search algorithm. 2. Table S1. Characteristics of studies included in the systematic review. 3. Figure S1. Evaluation of randomized controlled trials. 4. Table S2. Results of meta-analysis comparison of SGLT-2i inhibitors and placebo. 5. Figure S2. The primary outcome of SGLT-2i and placebo on the all-cause mortality in T2DM subgroup. 6. Figure S3. The primary outcome of SGLT-2i and placebo on the Cardiovascular mortality in T2DM subgroup. 7. Figure S4. The primary outcome of SGLT-2i and placebo on the HHF in T2DM subgroup. 8. Figure S5. The primary outcome of SGLT-2i and placebo on the all-cause mortality in heart failure subgroup. 9. Figure S6. The primary outcome of SGLT-2i and placebo on the Cardiovascular mortality in heart failure subgroup. 10. Figure S7. The primary outcome of SGLT-2i and placebo on the HHF in heart failure subgroup. 11. Figure S8. The primary outcome of SGLT-2i and placebo on the all-cause mortality in individual drugs subgroup. 12. Figure S9. The primary outcome of SGLT-2i and placebo on the Cardiovascular mortality in individual drugs subgroup. 13. Figure S10. The primary outcome of SGLT-2i and placebo on the HHF in individual drugs subgroup. 14. Figure S11. The adverse reactions of SGLT-2i and placebo on the ketoacidosis in individual drugs subgroup. 15. Table S3. Sensitivity analysis of acute renal failure. 16. Figure S12. The adverse reactions of SGLT-2i and placebo on the acute renal injury in individual drugs subgroup. 17. Table S4. Rankogram of the individual drugs. [file 12933_2021_1272_MOESM1_ESM.docx]

ADDITIONAL FILE

1. Search Algorithm.
2. Additional Table S1: Characteristics of studies included in the systematic review.
3. Additional Figure S1: Evaluation of randomized controlled trials.
4. Additional Table S2: Results of meta-analysis comparison of SGLT-2i inhibitors and placebo.
5. Additional Figure S2: The primary outcome of SGLT-2i and placebo on the all-cause mortality in T2DM subgroup.
6. Additional Figure S3: The primary outcome of SGLT-2i and placebo on the Cardiovascular mortality in T2DM subgroup.
7. Additional Figure S4: The primary outcome of SGLT-2i and placebo on the HHF in T2DM subgroup.
8. Additional Figure S5: The primary outcome of SGLT-2i and placebo on the all-cause mortality in heart failure subgroup.
9. Additional Figure S6: The primary outcome of SGLT-2i and placebo on the Cardiovascular mortality in heart failure subgroup.
10. Additional Figure S7: The primary outcome of SGLT-2i and placebo on the HHF in heart failure subgroup.
11. Additional Figure S8: The primary outcome of SGLT-2i and placebo on the all-cause mortality in individual drugs subgroup.
12. Additional Figure S9: The primary outcome of SGLT-2i and placebo on the Cardiovascular mortality in individual drugs subgroup.
13. Additional Figure S10: The primary outcome of SGLT-2i and placebo on the HHF in individual drugs subgroup.
14. Additional Figure S11: The adverse reactions of SGLT-2i and placebo on the ketoacidosis in individual drugs subgroup.
15. Additional Table S3: Sensitivity analysis of acute renal failure.
16. Additional Figure S12: The adverse reactions of SGLT-2i and placebo on the acute renal injury in individual drugs subgroup.
17. Additional Table S4: Rankogram of the individual drugs.

1.Search Algorithm:

**PubMed Search:**

(“Cardiovascular disease” [Mesh] OR “Cardiovascular disease” [tiab] OR “heart failure” [tiab] OR “Myocardial Infarction”[tiab]) OR “Atrial fibrillation”[tiab])AND (“Dapagliflozin”[tiab] OR “Empagliflozin”[tiab] OR “Canagliflozin”[tiab] OR “Ertugliflozin”[tiab] OR “SGLT2”[tiab] OR “Sodium-glucose co-transporter 2”[tiab])

**Embase Search:**

('Cardiovascular disease'/exp OR 'Cardiovascular disease': ab,ti OR ' heart failure ':ab,ti OR ' Myocardial Infarction ':ab,ti OR ' Atrial fibrillation':ab,ti) AND (Dapagliflozin:ab,ti OR Empagliflozin:ab,ti OR Ertugliflozin:ab,ti OR Canagliflozin:ab,ti)

**The Cochrane Library search strategy:**

#1 MeSH descriptor: [Cardiovascular Diseases] explode all trees

#2 Cardiovascular disease OR heart failure OR Myocardial Infarction OR Atrial fibrillation

#3 #1 or #2

#4 Dapagliflozin OR Empagliflozin OR Canagliflozin OR Ertugliflozin OR SGLT2 OR Sodium-glucose co-transporter 2

#5 #3 and #4

**Web of Science search strategy:**

#1 (TS=(Cardiovascular disease OR heart failure OR Myocardial Infarction OR Atrial fibrillation))

#2 (TS=(Dapagliflozin OR Empagliflozin OR Canagliflozin OR Ertugliflozin OR SGLT2 OR Sodium-glucose co-transporter 2))

#3 #1 and #2

**ClinicalTrials.gov (function ‘Search for Studies’ was used):**

(Dapagliflozin OR Empagliflozin OR Canagliflozin OR SGLT2 OR Sodium-glucose co-transporter 2 OR Ertugliflozin) AND Cardiovascular disease

2. Additional Table S1: Characteristics of studies included in the systematic review.

| Study | Study design | Patients, n | Age | Male (%) | Follow-up (years) | Basic cardiovascular disease | SGLT-2/control group | With or without T2DM | Major clinical outcomes |
| --- | --- | --- | --- | --- | --- | --- | --- | --- | --- |
| Zinman 2015[12] | RCT | 7020 | ＜65yr:55.5%  ≥65yr:44.5% | 71.5 | 3.1 | Various | Empagliflozin/placebo | T2DM | All-cause mortality, cardiovascular death, hospitalization for heart failure |
| Neal 2017[13] | RCT | 10142 | 63.3 | 64.2 | 3.6 | Various | Canagliflozin/placebo | T2DM | All-cause mortality, cardiovascular death, hospitalization for heart failure |
| Kosiborod 2017[14] | RCT | 320 | 64 | 62.8 | 1 | Heart failure | Dapagliflozin/placebo | T2DM | All-cause mortality |
| Perkovic 2019[15] | RCT | 4401 | 63 | 66.1 | 2.62 | Various | Canagliflozin/placebo | T2DM | All-cause mortality, cardiovascular death, hospitalization for heart failure |
| Furtado 2019[16] | RCT | 17160 | 64 | 62.6 | 4.2 | MI，ASCVD | Dapagliflozin/placebo | T2DM | All-cause mortality, cardiovascular death, hospitalization for heart failure |
| McMurray 2019[17] | RCT | 4744 | 66.3 | 76.6 | 1.5 | Heart failure | Dapagliflozin/placebo | no T2DM | All-cause mortality, cardiovascular death, hospitalization for heart failure |
| Nassif 2019[18] | RCT | 263 | 61.3 | 73.4 | 0.23 | HFrEF | Dapagliflozin/placebo | no T2DM | All-cause mortality, cardiovascular death, hospitalization for heart failure |
| Bhatt 2020[19] | RCT | 1222 | 69.5 | 66.3 | 0.75 | HFrEF and HFpEF | Sotagliflozin/placebo | T2DM | All-cause mortality, cardiovascular death, hospitalization for heart failure |
| Cannon 2020[20] | RCT | 8246 | 64.4 | 70.0 | 3.5 | ASCVD | Ertugliflozin/placebo | T2DM | All-cause mortality, cardiovascular death, hospitalization for heart failure |
| Packers 2020[21] | RCT | 3730 | 66.8 | 76.1 | 0.31 | HFrEF | Empagliflozin/placebo | no T2DM | All-cause mortality, cardiovascular death, hospitalization for heart failure |

Abbreviations: T2DM: type 2 diabetes; HFrEF: heart failure with reduced ejection fraction; HFpEF: heart failure with preserved ejection fraction; RCT: randomised controlled trial; MI: myocardial infarction; ASCVD: atherosclerotic cardiovascular disease.

3. Additional Figure S1: Evaluation of randomized controlled trials. (A: Risk of bias graph; B: Risk of bias summary)


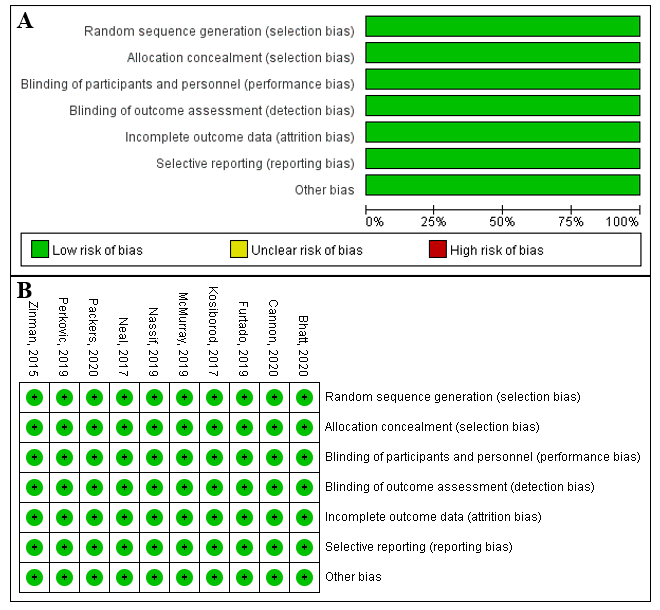


4. Additional Table S2: Results of meta-analysis comparison of SGLT-2i inhibitors and placebo.

| Classification | | Outcomes | No of Trials | SGLT-2i patients | Placebo patients | RR/MD  (95% CI) | p value | Study heterogeneity | | | |
| --- | --- | --- | --- | --- | --- | --- | --- | --- | --- | --- | --- |
|  |  |  |  |  |  |  |  | χ^2^ | I^2^, % | p value | |
| Cardiovascular outcome | | All-cause mortality | 10 | 25108 | 18574 | 0.86  (0.81 to 0.91) | <0.00001 | 9.03 | 0 | 0.43 | |
|  |  | Cardiovascular mortality | 9 | 24933 | 18421 | 0.85  (0.79 to 0.92) | <0.0001 | 10.75 | 26 | 0.22 | |
|  |  | Hospitalization for heart failure | 9 | 24959 | 18436 | 0.69  (0.64 to 0.74) | <0.00001 | 4.87 | 0 | 0.77 | |
|  |  | Hospitalization for heart failure or cardiovascular death | 7 | 23315 | 16813 | 0.78  (0.73 to 0.83) | <0.00001 | 7.39 | 19 | 0.29 | |
|  |  | major adverse cardiovascular event | 5 | 18348 | 11773 | 0.89  (0.83 to 0.95) | 0.0003 | 5.25 | 24 | 0.26 | |
|  |  | Fatal or nonfatal myocardial infarction | 4 | 17753 | 11230 | 0.90  (0.82 to 0.99) | 0.03 | 4.41 | 32 | 0.22 | |
|  |  | Nonfatal myocardial infarction | 4 | 16112 | 9559 | 0.91  (0.81 to 1.02) | 0.12 | 4.95 | 39 | 0.18 | |
|  |  | Nonfatal stroke | 3 | 15981 | 9427 | 0.99  (0.85 to 1.15) | 0.92 | 4.05 | 48 | 0.15 | |
|  |  | Fatal or nonfatal stroke | 5 | 17869 | 11379 | 0.98  (0.86 to 1.11) | 0.72 | 5.04 | 21 | 0.28 | |
| Renal outcomes | | Composite renal outcome | 6 | 26713 | 20187 | 0.72  (0.66 to 0.78) | <0.00001 | 6.04 | 34 | 0.20 | |
|  |  | Acute renal failure | 4 | 13021 | 9197 | 1.00  (0.90 to 1.11) | 0.99 | 18.34 | 84 | 0.0004 | |
|  |  | Acute renal injury | 7 | 20850 | 14274 | 0.80  (0.68 to 0.93) | 0.004 | 5.35 | 0 | 0.50 | |
| Safety outcomes | **Adverse endocrine outcomes** | Hypoglycemic | 6 | 18645 | 12074 | 1.00  (0.96 to 1.05) | 0.87 | 5.43 | 8 | 0.37 | |
|  |  | Diabetic ketoacidosis | 6 | 20674 | 14122 | 3.65  (1.83 to 7.27) | 0.0002 | 3.14 | 0 | 0.53 | |
|  | **Adverse events** | Adverse events leading to discontinuation | 6 | 18645 | 12074 | 1.00  (0.94 to 1.07) | 0.99 | 7.86 | 36 | 0.16 |  |
|  |  | Any adverse event | 5 | 12682 | 7556 | 0.98  (0.97 to 0.99) | 0.004 | 5.78 | 31 | 0.22 |  |
|  |  | Serious adverse event | 6 | 18477 | 11903 | 0.91  (0.88 to 0.93) | <0.00001 | 7.79 | 36 | 0.17 |  |
|  | **Adverse infection outcomes** | Urinary tract infection | 4 | 16146 | 9574 | 1.08  (1.01 to 1.15) | 0.02 | 4.88 | 39 | 0.18 |  |
|  |  | Male Genital infection | 3 | 15975 | 9425 | 3.35  (2.90 to 3.87) | <0.00001 | 1.16 | 0 | 0.56 |  |
|  |  | Mycotic genital infection in women | 3 | 15975 | 9425 | 3.85  (3.43 to 4.32) | <0.00001 | 1.76 | 0 | 0.41 |  |
|  |  | Acute pancreatitis | 3 | 13488 | 9289 | 1.12  (0.64 to 1.98) | 0.69 | 1.45 | 0 | 0.48 |  |
|  | **Others** | Bone fracture | 5 | 20543 | 13990 | 1.11  (0.99 to 1.23) | 0.08 | 5.24 | 24 | 0.26 |  |
|  |  | Amputation | 4 | 15856 | 11657 | 1.42  (1.18 to 1.71) | 0.0002 | 4.41 | 32 | 0.22 |  |
|  |  | Hypovolemia | 6 | 18645 | 12074 | 1.22  (1.11 to 1.33) | <0.0001 | 9.15 | 45 | 0.10 |  |

5. Additional Figure S2: The primary outcome of SGLT-2i and placebo on the all-cause mortality in T2DM subgroup.


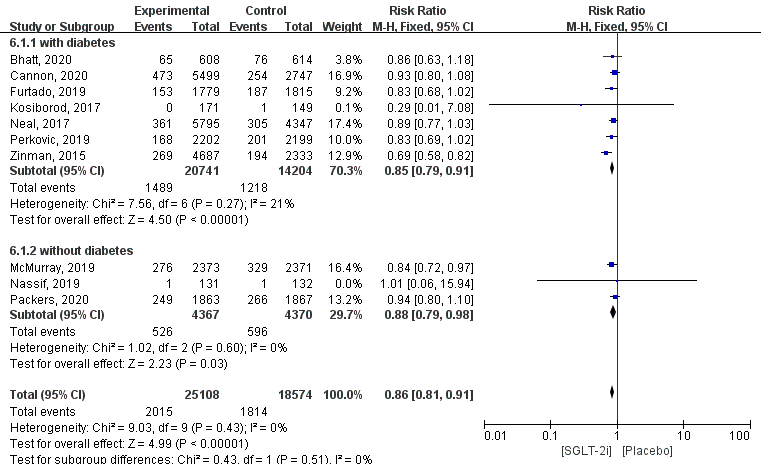


6. Additional Figure S3: The primary outcome of SGLT-2i and placebo on the Cardiovascular mortality in T2DM subgroup.


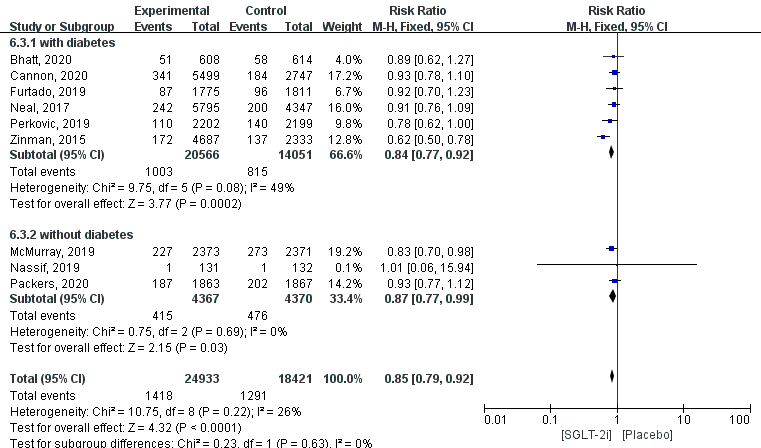


7. Additional Figure S4: The primary outcome of SGLT-2i and placebo on the HHF in T2DM subgroup.


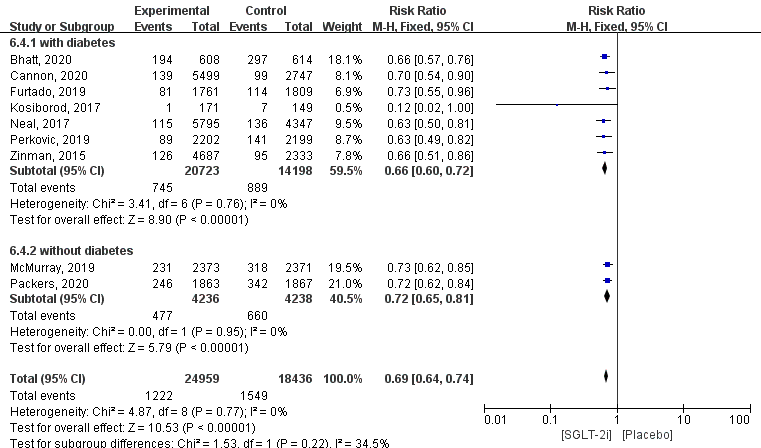


8.Additional Figure S5: The primary outcome of SGLT-2i and placebo on the all-cause mortality in heart failure subgroup.


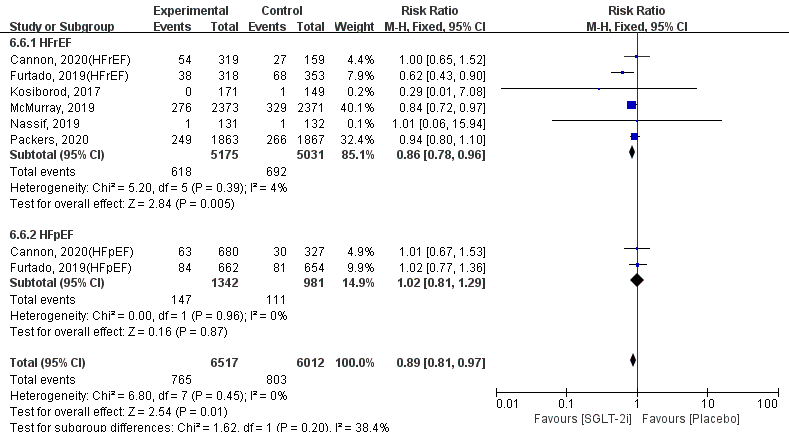


9. Additional Figure S6: The primary outcome of SGLT-2i and placebo on the Cardiovascular mortality in heart failure subgroup.


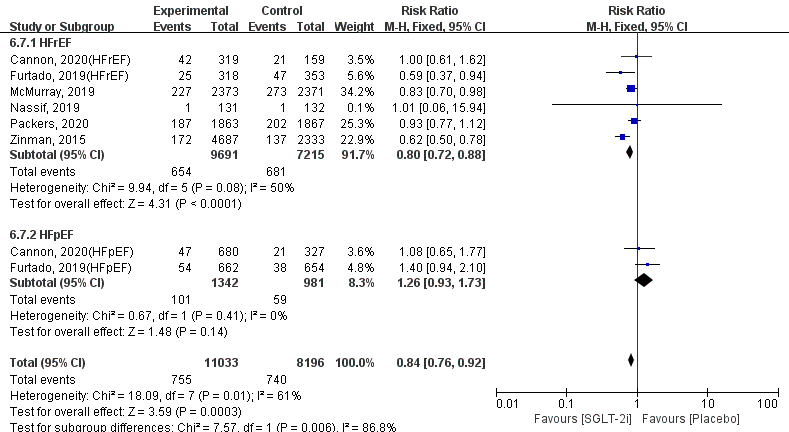


10. Additional Figure S7: The primary outcome of SGLT-2i and placebo on the HHF in heart failure subgroup.


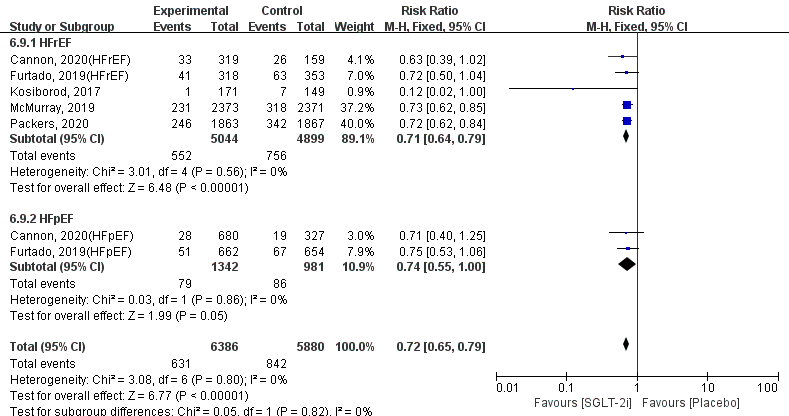


11. Additional Figure S8: The primary outcome of SGLT-2i and placebo on the all-cause mortality in individual drugs subgroup.


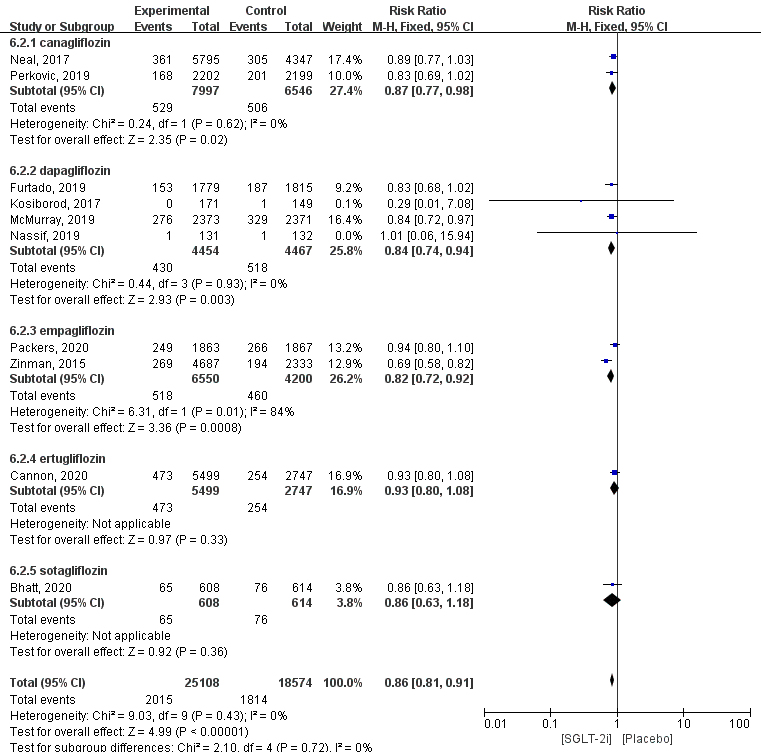


12. Additional Figure S9: The primary outcome of SGLT-2i and placebo on the Cardiovascular mortality in individual drugs subgroup.


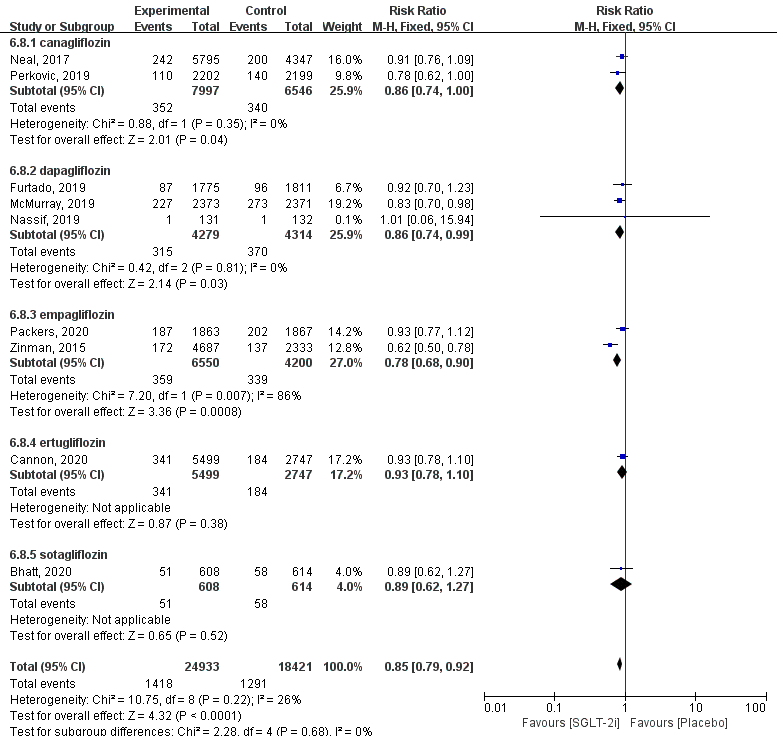


13. Additional Figure S10: The primary outcome of SGLT-2i and placebo on the HHF in individual drugs subgroup.


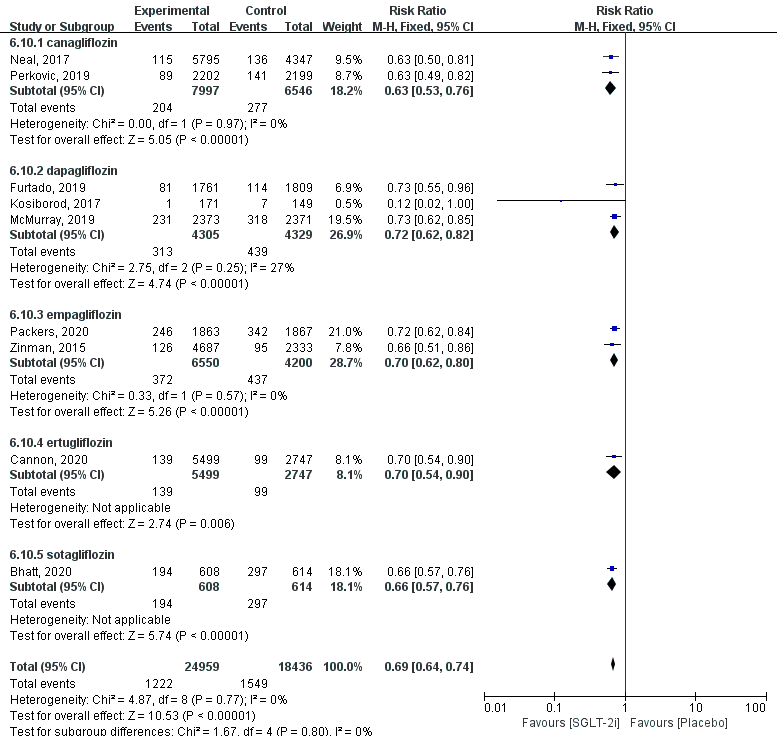


14. Additional Figure S11: The adverse reactions of SGLT-2i and placebo on the ketoacidosis in individual drugs subgroup.


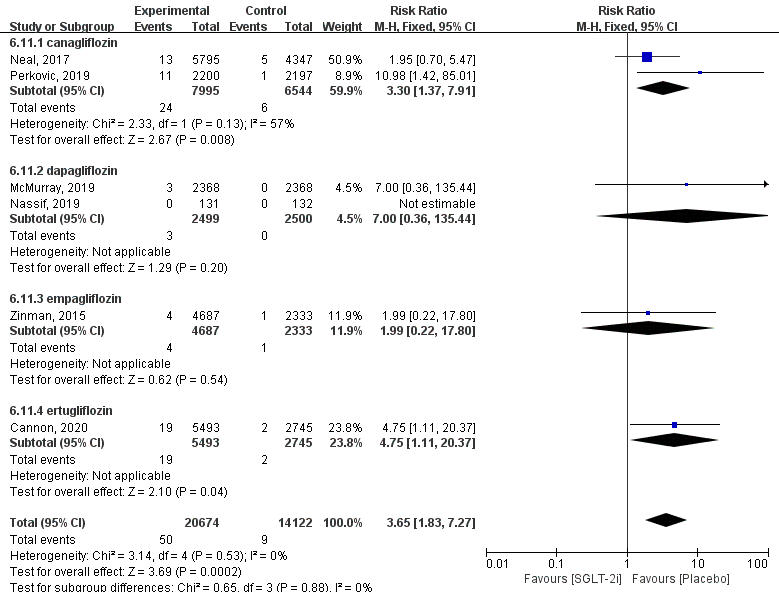


15. Additional Table S3: Sensitivity analysis of acute renal failure.

| **Excluded trial** | ***I*^2^** | **RR (95% CI)** | ***P* value** |
| --- | --- | --- | --- |
| **Zanman 2015** | 78.4% | 1.08 (0.96, 1.22) | 0.20 |
| **Neal 2017** | 80.6% | 0.89 (0.78, 1.03) | 0.12 |
| **Kosiborod 2017** | 74.8% | 0.97 (0.88, 1.08) | 0.61 |
| **McMurray 2019** | 86.4% | 1.03 (0.91, 1.15) | 0.51 |

Abbreviations: RR, Risk ratio; 95% CI, 95% confidence interval.

1. Additional Figure S12: The adverse reactions of SGLT-2i and placebo on the acute renal injury in individual drugs subgroup.


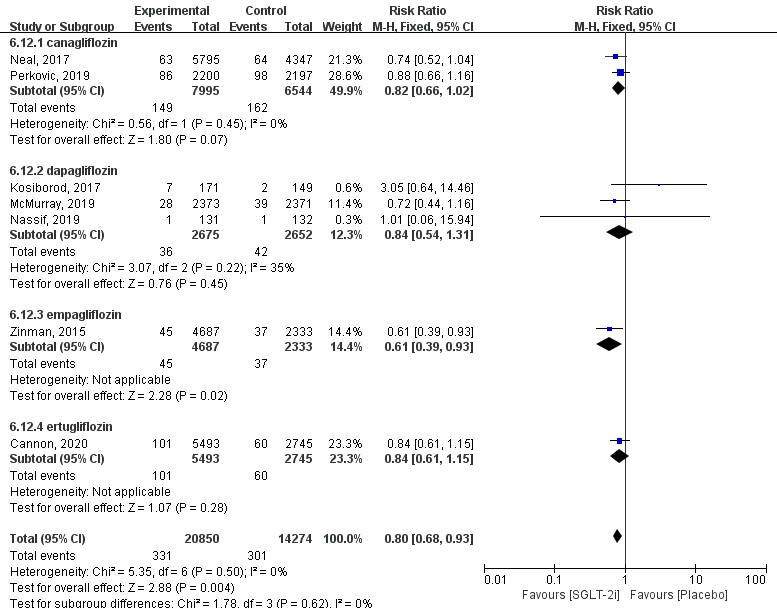


17.Additional Table S4: Rankogram of the individual drugs

| Rank | All-cause morality | Cardiovascular morality | HHF | Acute renal injury | Ketoacidosis |
| --- | --- | --- | --- | --- | --- |
| 1 | Dapagliflozin (22%) | Empagliflozin (23%) | Sotagliflozin (7%) | Empagliflozin (22%) | Placebo (11%) |
| 2 | Canagliflozin (36%) | Canagliflozin (43%) | Canagliflozin (34%) | Canagliflozin (44%) | Empagliflozin (40%) |
| 3 | Sotagliflozin (38%) | Dapagliflozin (45%) | Empagliflozin (48%) | Ertugliflozin (50%) | Canagliflozin (50%) |
| 4 | Ertugliflozin (57%) | Sotagliflozin (51%) | Ertugliflozin (54%) | Dapagliflozin (62%) | Ertugliflozin (54%) |
| 5 | Empagliflozin (61%) | Ertugliflozin (59%) | Dapagliflozin (58%) | Placebo (72%) | Dapagliflozin (95%) |
| 6 | Placebo (85%) | Placebo (80%) | Placebo (99%) | NA | NA |
